# An evaluation of a national mass media campaign to raise public awareness of possible lung cancer symptoms in England in 2016 and 2017

**DOI:** 10.1038/s41416-021-01573-w

**Published:** 2021-10-30

**Authors:** Susan Ball, Chris Hyde, Willie Hamilton, Chloe J. Bright, Carolynn Gildea, Kwok F. Wong, Lizz Paley, Helen L. Hill, Vivian Mak, Jodie Moffat, Lucy Elliss-Brookes

**Affiliations:** 1 NIHR ARC South West Peninsula (PenARC), University of Exeter Medical School, University of Exeter, Exeter, EX1 2LU UK; 2 University of Exeter Medical School, University of Exeter, Exeter, EX1 2LU UK; 3 National Cancer Registration and Analysis Service, Public Health England, Wellington House, London, SE1 8U UK; 4 Cancer Research UK, 2 Redman Place, London, E20 1JQ UK

**Keywords:** Translational research, Epidemiology

## Abstract

**Background:**

A two-phase ‘respiratory symptoms’ mass media campaign was conducted in 2016 and 2017 in England raising awareness of cough and worsening shortness of breath as symptoms warranting a general practitioner (GP) visit.

**Method:**

A prospectively planned pre–post evaluation was done using routinely collected data on 15 metrics, including GP attendance, GP referral, emergency presentations, cancers diagnosed (five metrics), cancer stage, investigations (two metrics), outpatient attendances, inpatient admissions, major lung resections and 1-year survival. The primary analysis compared 2015 with 2017. Trends in metrics over the whole period were also considered. The effects of the campaign on awareness of lung cancer symptoms were evaluated using bespoke surveys.

**Results:**

There were small favourable statistically significant and clinically important changes over 2 years in 11 of the 15 metrics measured, including a 2.11% (95% confidence interval 1.02–3.20, *p* < 0.001) improvement in the percentage of lung cancers diagnosed at an early stage. However, these changes were not accompanied by increases in GP attendances. Furthermore, the time trends showed a gradual change in the metrics rather than steep changes occurring during or after the campaigns.

**Conclusion:**

There were small positive changes in most metrics relating to lung cancer diagnosis after this campaign. However, the pattern over time challenges whether the improvements are wholly attributable to the campaign. Given the importance of education on cancer in its own right, raising awareness of symptoms should remain important. However further research is needed to maximise the effect on health outcomes.

## Background

Lung cancer remains a very important health problem globally, including the United Kingdom, where it represented 13% of newly diagnosed cancers cases in 2016 [1]. It is the second most common cancer in both genders and is the most common cause of UK cancer death, with 18,810 men and 16,338 women dying from it in 2017 [2]. Although there is evidence of steady improvement over time, lung cancer survival remains poor, particularly relative to the three other most common cancers [3]. Changes in lung cancer incidence over time are complex, generally decreasing in men, but increasing in women, reflecting historical smoking patterns. The expanding and ageing UK population is predicted to increase lung cancer incidence, as it is highly age-dependent. The net effect is that UK lung cancer numbers are currently slowly increasing.

Survival in lung cancer is related to the stage at diagnosis. Stage 1 has a 5-year survival of 56.6%, whereas Stage 4 has a 5-year survival of 2.9%. However, most lung cancers are present at Stage 4 [4]. Furthermore, one-third of lung cancers present as an emergency, with the complication precipitating the emergency presentation bringing additional mortality [5]. These facts suggest an opportunity to improve the outcome of lung cancer by identifying it earlier. Screening with low-dose computed tomography (CT) currently shows great promise based on the USA [6] and recent European trials [7–9].

Promoting public awareness of early symptoms is a further secondary prevention approach. The rationale and general framework for improving outcomes and advancing diagnosis in cancer are laid out in the National Awareness and Early Diagnosis Initiative (NAEDI) models [10, 11]. The theoretical model for lung cancer is that patients would recognise symptoms of possible cancer earlier (including symptoms previously considered as ‘normal’ for the individual). The increased recognition might act during the awareness campaign or for future symptoms. It may also be mediated through the individuals’ families. Symptom recognition would lead to attendance at primary care, which would investigate possible lung cancer. This pathway requires several steps to improve outcomes. Individuals newly recognising the potential implications of their symptoms must act by entering healthcare; general practitioners (GPs) must be receptive to more patients presenting with low-risk symptoms and there needs to be sufficient investigative capacity in the health system to accommodate increased numbers in a timely fashion. This expedited process may improve outcomes through stage shift, plus by a reduction in emergency presentation, independent of any stage shift.

In 2012 the first national (England) public awareness campaign relating to possible lung cancer focused on the symptom of cough persisting for more than 3 weeks. It followed a successful regional pilot and was evaluated by comparing metrics during and immediately after the 8-week mass media campaign with the same period a year before [12]. The secular change was estimated by considering the change in a ‘control’ period, immediately before the mass media campaign, with the same period a year before. The 3-monthly lung cancer incidence increased by 9.1% in the ‘campaign’ 1-year pre–post change, but only by 1.5% for the ‘control’ 1-year pre–post change. A stage shift was identified, with an increase in Stage 1 and a fall in Stage 4 at diagnosis in the ‘campaign’ 1-year pre–post change. There was a consistent pattern of changes in other metrics favouring a positive impact of the campaign, including increased awareness of lung cancer symptoms, presentations to primary care, GP referrals for imaging and GP referrals for suspected cancer. These led to the conclusion that the campaign had been effective.

Based on the effectiveness of the 2012 campaign, a further campaign was planned for 2016 and 2017, modified to include shortness of breath as well as cough, with the intention of improving identification of not just lung cancer but other serious respiratory and cardiovascular disease too. An evaluation similar to that for the cough-only campaigns was pre-planned and led by an independent team who finalised the analytical framework. This paper reports this evaluation.

## Materials and methods

The ‘campaign’ was part of the Be Clear on Cancer programme, led by Public Health England, aiming to improve the early diagnosis of cancer by raising public awareness of symptoms and signs of cancer and encouraging people to attend their GP without delay. The national mass media campaign ran across England from 14 July 2016 to 16 October 2016 (Phase 1) [13 weeks] and from 18 May 2017 to 31 August 2017 (Phase 2) [16 weeks] using the ‘Be Clear on Cancer’ brand with the core messages:

If you’ve had a cough for three weeks or more, it could be a sign of lung disease, including cancer. Finding it early makes it more treatable. So don’t ignore it, tell your doctor.

and

If you get out of breath doing things you used to be able to do, it could be a sign of lung or heart disease, or even cancer. Finding it early makes it more treatable. So don’t ignore it, tell your doctor.

The campaign activity was aimed at men and women aged 50 years and over. The ‘Be Clear on Cancer’ website provides more detail [13].

### Data collection and analysis

Data were collected and analysed for the set of metrics described below, to represent the patient pathway from symptom awareness, through to diagnosis, stage, treatment and survival. The main analyses explored changes in metrics following the campaign launch, by considering Phase 1 (in 2016) and Phase 2 (2017) as a single campaign, comparing the period during and shortly after Phase 2 (analysis period), to the equivalent period before Phase 1 (comparison period). Further analyses explored changes in metrics following the launch of Phase 1 of the campaign, by comparing the period during and shortly after Phase 1 (analysis period), to the equivalent period before Phase 1 (comparison period). The exact time periods varied slightly across metrics accommodating the expectation that each metric would be impacted at different times (e.g. GP attendances should change very soon after the start of the campaign, whereas cancer diagnoses would change after a few weeks). The analysis and comparison periods used for each metric are detailed in Table 1.

**Table 1:** Analysis and comparison periods used for each metric in main analyses and further analyses.

Metric	Main analyses: impact of Phase 1 and 2 combined		Further analyses: impact of Phase 1	
	Comparison period^a^	Analysis period^a^	Comparison period^a^	Analysis period^a^
GP attendances (people aged ≥50)^b^	18 May to 6 September 2015	22 May to 10 September 2017	18 July to 30 October 2015	13 July to 25 October 2016
TWW referrals	May to September 2015	May to September 2017	July to November 2015	July to November 2016
Cancers diagnoses resulting from a TWW referral^c^	May to September 2015	May to September 2017	July to November 2015	July to November 2016
TWW referrals resulting in a cancer diagnosis (conversion)^c^	May to September 2015	May to September 2017	July to November 2015	July to November 2016
New cancers recorded in CWT database^d^	June to October 2015	June to October 2017	August to December 2015	August to December 2016
Cancers diagnosed recorded in CWT database from TWW referral (detection)^d^	June to October 2015	June to October 2017	August to December 2015	August to December 2016
Emergency presentations^e^	May to October 2015	May to October 2017	July to December 2015	July to December 2016
Cancers diagnosed^f^	25 May to 25 October 2015	29 May to 29 October 2017	20 July to 13 December 2015	25 July to 18 December 2016
Early stage at diagnosis	25 May to 25 October 2015	29 May to 29 October 2017	20 July to 13 December 2015	25 July to 18 December 2016
Diagnostics in secondary care	May to October 2015	May to October 2017	July to December 2015	July to December 2016
Echocardiograms	May to September 2015	May to September 2017	July to November 2015	July to November 2016
Outpatient attendances to cardiac or respiratory	25 May to 25 October 2015	29 May to 29 October 2017	13 July to 27 December 2015	18 July 2016 to 1 January 2017
Inpatient admissions to cardiac or respiratory	25 May to 25 October 2015	29 May to 29 October 2017	13 July to 20 December 2015	11 July to 18 December 2016
Major resections	June to October 2015	June to October 2017	July to December 2015	July to December 2016
Survival	1 June to 31 October 2015	1 June to 31 October 2017	28 July to 11 December 2015	28 July to 11 December 2016

*GP* general practice, *TWW* 2-week wait, *CWT* cancer waiting time.

^a^Exact dates within months are given where data were weekly proportions or counts; otherwise, data were monthly proportions or counts.

^b^Visits with respiratory symptoms.

^c^Based on ‘date first seen’ in CWT database.

^d^Based on ‘treatment start date’ in CWT database.

^e^From inpatient Hospital Episode Statistics.

^f^From cancer registration database.

#### Awareness of lung cancer symptoms

Responses to key questions relating to the campaign’s core messages were obtained through surveys conducted by an independent organisation immediately before, during and immediately after each of the two phases of the campaign. Only the results pre the first phase and post the second were used in our analysis. Surveys were conducted online in those aged 50–69 years and face to face in those aged 70+ years in 2017; 2016 data were adjusted post hoc to mirror this pattern, ignoring data collected face to face on those aged 50–69 years. Data collected were combined and weighted to be nationally representative on the basis of age, gender, socioeconomic status and geographic region. The questions deemed most relevant to the linkage between awareness and likelihood of action were the focus of the analysis, which compared before Phase 1 (24 June 2016 to 5 July 2016) with the end of Phase 2 (19 September 2017 to 2 October 2017). The complete survey was an extensive examination of the effectiveness of the campaign in meeting its stated objectives. It included questions on reaction to breathlessness or persistent cough symptoms; agreement of symptoms being a sign of something more serious; recent exposure to adverts, publicity or other types of information; the meaning of breathlessness or persistent cough; agreement with statements on various health conditions; reasons for putting off going to see the GP/doctor; agreement with statements concerning doctor waste of time/judgement/worry; agreement with statements about the advertising; recognition of aspects of the campaign; knowledge of persons with heart and lung conditions; and aspects of health and lifestyle.

#### GP attendances

Data on GP attendances by those aged 50 years and above for respiratory symptoms (cough, breathlessness, other respiratory symptoms) were sourced from The Health Improvement Network (THIN) [14]. Data were grouped into weeks and adjusted to account for bank holidays.

#### Urgent GP referrals for suspected lung cancer (2-week wait (TWW) referrals) and cancers diagnosed from TWW referral

Data on GP referrals for suspected lung cancer, new lung cancer cases that resulted from a TWW referral and the percentage of TWW referrals resulting in a diagnosis of lung cancer (conversion rate) were sourced from the National Cancer Waiting Times Monitoring Dataset [15]. Data were monthly, with referrals and diagnoses reported in the month that the patient was first seen. Lung cancers were defined as an International Statistical Classification of Diseases and Related Health Problems 10th Revision (ICD-10) diagnosis code of C33-C34, C37-C39 or C45.

#### Cancer diagnoses recorded in the cancer waiting time (CWT) database

Data on the number of lung cancer diagnoses recorded in the CWT database and on the percentage of new CWT database recorded lung cancer diagnoses, which resulted from a TWW referral (detection rate), were taken from the National Cancer Waiting Times Monitoring Dataset [15]. Data were monthly and diagnoses were reported in the month of first treatment. Lung cancer cases were defined as those with an ICD-10 diagnosis code of C33-C34, C37-C39 or C45.

#### Emergency presentations

The Hospital Episode Statistics (HES) emergency presentation metric was calculated from inpatient data, using the methodology set out in the cancer outcomes metric specification [16]. We report monthly percentages of lung cancer diagnoses first presenting as an emergency.

#### Cancers diagnosed and early stage at diagnosis

Data on the weekly number of newly diagnosed cases of lung cancer (ICD-10 of C33-34) were extracted from the National Cancer Registration Dataset in England [17]. Of those cases that were staged, the weekly percentages of early-stage (Stages 1, 2 and 3a) cases were calculated.

#### Diagnostics in secondary care

Data on the monthly number of X-ray and CT scans conducted for suspected lung cancer by GPs were obtained from the Diagnostic Imaging Dataset held on the NHS Digital’s iView system. Data were restricted to X-ray and CT scans referred via GP surgeries.

#### Echocardiograms

Data on the monthly number of echocardiograms performed were sourced from the NHS Monthly Diagnostic Waiting Times and Activity dataset [18].

#### Outpatient attendances and inpatient admissions

Data on weekly numbers of outpatient attendances seen under cardiac and respiratory services (cardiology, respiratory and general medicine) and on weekly numbers of inpatient admissions with either heart failure, chronic obstructive pulmonary disease or dyspnoea were taken from the HES dataset.

#### Major resections for lung cancer

Data were extracted from the National Cancer Registration Dataset in England. Monthly percentages of patients with lung cancer who had a major resection within 6 months of their diagnosis were calculated.

#### Survival

Data on the time to death or last follow-up (up to 1 year) of patients diagnosed with lung cancer during the analysis and comparison periods were provided by NHS Digital, in cohorts defined using the National Cancer Registration Dataset.

##### Analysis

For survey questions relating to an agreement of symptoms being a sign of something more serious, the percentages of respondents answering ‘agree strongly’ or ‘agree’, from possible response options of ‘agree strongly’, ‘agree’, ‘disagree’, ‘disagree strongly’ and ‘don’t know’, were compared between analysis and comparison periods using the two-sample test of proportions. Similarly, the percentages of respondents answering that they would visit their GP if they were breathless doing things they could usually do, and if they had a cough lasting 3 weeks or longer, were compared between periods. Results are reported as numbers and percentages in each period and the absolute difference in percentages between the two periods, with 95% confidence interval (CI) and *p* value.

Where metrics were measured using weekly or monthly percentages, these were aggregated over the analysis and comparison periods to give a single percentage for each period, and the two-sample test of proportions was used to test for any difference between the periods. Results are reported as numbers and percentages in each period and the absolute difference in percentages between the two periods, with 95% CI and *p* value. Where data were weekly or monthly counts, these were compared using negative binomial or Poisson regression, with a single explanatory variable coded as 0 and 1 for the comparison and analysis period. For GP attendances, the number of practices associated with weekly numbers of attendances was included as an exposure in the fitted model. Results are reported as the total count in each period and the estimated rate ratio (analysis period relative to comparison period), with 95% CI and *p* value. Survival data were compared using Cox regression, with time to death or end of follow-up (1 year) as the outcome, and reported as median survival and the estimated hazard ratio (analysis period relative to comparison period), with 95% CI and *p* value.

Analyses were based on people of all ages, except for GP attendances and survival, which used people aged ≥50 years. As <5% of lung cancers are diagnosed below the age of 50 years, this decision (for operational reasons) will have made no important difference to the results. For metrics where the main analysis used data on people of all ages, sensitivity analyses examined those aged ≥50 years, where possible.

Weekly or monthly data for the 3-year period 2015–2017 were graphed and examined qualitatively for evidence of phasic changes occurring after either or both of the two phases of the campaign.

All tests were conducted under a two-sided approach, with no adjustment for multiple testing. Analyses were carried out in Stata 16 [19] and R [20].

## Results

Results from the analyses of survey questions used to assess awareness of lung cancer symptoms are presented in Table 2.

**Table 2:** Public awareness pre–Phase 1 and post-Phase 2 survey results for key questions relating to the campaign’s core messages.

Metric	Comparison period (pre-Phase 1) *N* = 897^b^	Analysis period (post Phase 2) *N* = 833	Absolute difference in percentage (95% CI)	*P* value^a^
Agreement of symptoms being a sign of something more serious^c^, *n* (%)				
Getting out of breath doing things you used to be able to do	692 (77.1)	685 (82.2)	5.1% (1.3–8.9)	0.009
Coughing regularly for 3 weeks or more	731 (81.5)	733 (88.0)	6.5% (3.1–9.9)	<0.001
Coughing up blood	755 (84.2)	759 (91.1)	6.9% (3.9–10.0)	<0.001
What would you try if you were breathless doing things you can usually do, *n* (%)				
Visit your GP	554 (61.8)	448 (53.8)	−8.0% (−12.6 to −3.3)	<0.001
What would you try if you had a cough lasting 3 weeks or more, *n* (%)				
Visit your GP	676 (75.4)	568 (68.2)	−7.2% (−11.4 to −2.9)	<0.001

^a^*P* value from a test of two proportions.

^b^Adjusted numbers from Kantar.

^c^Those answering ‘agree’ or ‘strongly agree’ from possible response options: ‘strongly disagree’, ‘disagree’, ‘agree’, ‘strongly agree’ and ‘don’t know’.

There was an increase in awareness of symptoms targeted by the campaign: getting out of breath (difference in the percentage of respondents answering ‘agree strongly’ or ‘agree’ (analysis - comparison period) 5.1%, 95% CI 1.3–8.9); coughing regularly for 3 weeks (difference in percentage answering ‘agree strongly’ or ‘agree’ (analysis - comparison period) 6.5%, 95% CI 3.1–9.9). There was also a significant increase in awareness of the symptom coughing up blood, which was not a target of the campaign (difference in percentage (analysis - comparison period) 6.9%, 95% CI 3.9–10.0). However, there was a fall in the likelihood of the respondents saying they would visit their GP if these symptoms did occur: getting out of breath (difference in percentage (analysis - comparison period) −8.0%, 95% CI −12.6 to −3.3); coughing lasting 3 weeks or more (difference in percentage −7.2%, 95% CI −11.4 to −2.9).

Results from the main analyses of the routine data are detailed in Table 3. All changes refer to the period of the second phase of the campaign, compared to the same period before the first phase.

**Table 3:** Results from main analyses: changes in metrics across the whole campaign (Phases 1 and 2 combined).

Metric	Comparison period (pre-Phase 1)	Analysis period (during/post Phase 2)	Statistic	Estimate (95% CI)	*P* value
GP attendances (people aged ≥50)^a^	41,967 across 4367 practices	22,282 across 2419 practices	Rate ratio	0.96 (0.90–1.03)	0.2
TWW referrals	24,106	26,522	Rate ratio	1.10 (1.02–1.19)	0.02
Cancer diagnoses resulting from a TWW referral^b^	4488	4523	Rate ratio	1.01 (0.95–1.07)	0.8
TWW referrals resulting in a cancer diagnosis (conversion rate: %)^b^	18.62 (4488 out of 24,106)	17.05 (4523 out of 26,522)	Difference in percentage	−1.56% (−2.23 to −0.90%)	<0.001
New cancers recorded in CWT database^c^	12,804	13,703	Rate ratio	1.07 (1.01–1.13)	0.02
Cancers diagnosed recorded in CWT database from TWW referral (detection rate: %)^c^	38.47 (4926 out of 12,804)	34.57 (4737 out of 13,703)	Difference in percentage	−3.90% (−5.06 to −2.74%)	<0.001
Emergency presentations^d^	4685 out of 13,101 (35.76%)	4,639 out of 13,533 (34.28%)	Difference in percentage	−1.48% (−2.63 to −0.34%)	0.01
Cancers diagnosed^e,f^	16,370.75	16,944	Rate ratio	1.04 (1.01–1.06)	0.02
Early stage at diagnosis^e^	5776.75 early from 15,040 staged (38.41%)	6423.25 early from 15,851 staged (40.52%)	Difference in percentage	2.11% (1.02–3.20%)	<0.001
Diagnostics in secondary care: X-rays and CT scans	921,790 images	1,049,815 images	Rate ratio	1.14 (1.06–1.22)	<0.001
Echocardiograms	566,935 echocardiograms	631,283 echocardiograms	Rate ratio	1.11 (1.05–1.18)	<0.001
Outpatient attendances to cardiac or respiratory	4,026,991 attendances	4,263,144 attendances	Rate ratio	1.06 (1.04–1.08)	<0.001
Inpatient admissions to cardiac or respiratory	389,464 admissions	427,838 admissions	Rate ratio	1.10 (1.06–1.14)	<0.001
Major resections	2389 out of 15,900 (15.03%)	2754 out of 16,220 (16.98%)	Difference in percentage	1.95 (1.15– 2.76%)	<0.001
Survival (people aged ≥50)	Median survival 217 days	Median survival 231 days	Hazard ratio	0.95 (0.92–0.98)	<0.001

*GP* general practice, *TWW* 2-week wait, *CWT* cancer waiting time.

^a^Visits with respiratory symptoms.

^b^Based on ‘date first seen’ in CWT database.

^c^Based on ‘treatment start date’ in CWT database.

^d^From inpatient Hospital Episode Statistics.

^e^Where numbers of cases are not whole numbers, this is because weekly numbers of cases have been adjusted for bank holidays and summed over the period.

^f^From cancer registration database.

### GP attendances

The rate of presentations to GPs of patients aged ≥50 for respiratory symptoms was 4% lower (estimated rate ratio 0.96, 95% CI 0.90–1.03). Supplementary Fig. S1 confirms there was no upturn in attendances during the phases of the campaign.

### Urgent GP referrals for suspected lung cancer (TWW referrals)

There was an increase in urgent GP referrals for suspected lung cancer (estimated rate ratio 1.10, 95% CI 1.02–1.19). However, Supplementary Fig. S2 shows that this increase was not focused on the phases of the campaign. There was little evidence of a difference in the rate of cancer diagnoses resulting from a TWW referral (estimated rate ratio 1.01, 95% CI 0.95–1.07). The conversion rate decreased by 1.56% (95% CI −2.23 to −0.90).

### Cancer diagnoses recorded in the CWT database

The number of lung cancer diagnoses recorded in the CWT database increased (estimated rate ratio 1.07, 95% CI 1.01–1.13), although the percentage of recorded lung cancer diagnoses, which resulted from a TWW referral (detection rate), decreased by 3.9% (95% CI −5.1 to −2.7).

### Emergency presentations

The percentage of lung cancer diagnoses that first presented as an emergency was 1.48% lower (95% CI −2.63 to −0.34).

### Cancers diagnosed and early stage at diagnosis

The rate of lung cancers diagnosed was 4% higher (estimated rate ratio 1.04, 95% CI 1.01–1.06). The percentage of early stage at diagnosis also increased by 2.11% (95% CI 1.02–3.2).

### X-rays and CT scans and echocardiograms

The rate of imaging (X-ray or CT) and echocardiograms increased by 14% (estimated rate ratio 1.14, 95% CI 1.06–1.22) and 11% (estimated rate ratio 1.11, 95% CI 1.05–1.18), respectively.

### Outpatient attendances and inpatient admissions

The rate of outpatient attendances and inpatient admissions increased by 6% (estimated rate ratio 1.06, 95% CI 1.04–1.08) and 10% (estimated rate ratio 1.10, 95% CI 1.06–1.14), respectively.

### Major resections

The percentage of lung cancer diagnoses that resulted in a major resection was 1.95% higher (95% CI 1.15–2.76).

### Survival

The median survival was 231 and 217 days for patients diagnosed in the analysis and comparison period, respectively (HR 0.95, 95% CI 0.92–0.98, *p* < 0.001).

### Further analyses

The pattern of results was similar for the impact of Phase 1 alone (see Supplementary Table 1).

Results for sensitivity analyses based on people aged ≥50 years for the main and further analyses of were also similar to those for the analyses based on people of all ages (see Supplementary Tables 2 and 3).

### Trends

In general, apart from considerable background variability in many of the metrics, including pronounced seasonal effects, there were no consistent phasic patterns suggesting changes during or after each of the two phases of the campaign. The graphs suggest that the changes observed between pre and post the campaign were likely to be part of an underlying gradual trend (see Fig. 1 and Supplementary Figs. 1–10).

**Fig. 1:**
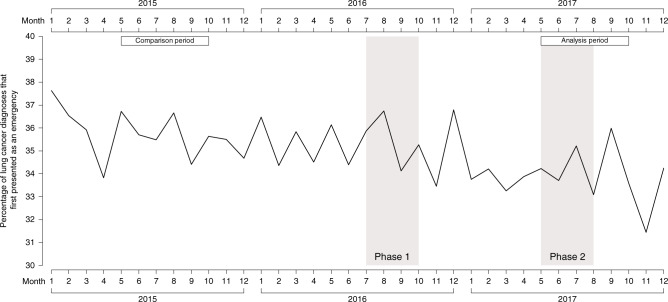
Emergency presentations. Monthly percentages of lung cancer diagnoses that first presented as an emergency, for the 3-year period (2015–2017).

## Discussion

The main findings are of small favourable statistically significant and clinically important changes over 2 years, from before the first phase of the campaign to after the second phase of it in 11 of the 15 metrics measured. For instance, the percentage of lung cancers presenting as an emergency fell by 1.48% (95% CI 2.63–0.34) and the percentage of cancers that were diagnosed at an early stage increased by 2.11% (95% CI 1.02–3.20). These are two key outcomes that underpin the improvement in median survival, with a hazard ratio of 0.95 (95% CI 0.92–0.98).

The four metrics that did not show this pattern were: the number of respiratory 2-week wait referrals, where there was an insignificant increase; GP attendances with a respiratory symptom that fell, although insignificantly (rate ratio 0.96; 95% CI 0.90–1.03); TWW referrals resulting in a cancer diagnosis (conversion rate: %), which fell statistically significantly; and cancers diagnosed recorded in CWT database from TWW referral (detection rate: %), which also fell statistically significantly. The time trends showed a gradual change in the metrics, rather than greater changes occurring during or after the campaign phases. The resulting profile was similar in the secondary and sensitivity analyses.

There was improved awareness of the key target symptoms in the surveys. However, there was also a fall in the percentages saying they would visit their GP if either of the target symptoms, cough for more than 3 weeks or shortness of breath, occurred. This last item is discordant with the other positive metrics.

The study has strengths and weaknesses. It benefits from being designed prospectively, with careful consideration of the nature and timing of the data collected. It also allowed for the campaign to be carefully described, particularly when it commenced and stopped. The metrics used (excluding awareness of lung cancer symptoms) were consistent throughout the period of observation, and their routine nature protected against manipulation. Data quality from our several sources is considered to be high, including Cancer Waiting Times, cancer registry data and the THIN primary care database [21, 22]. Furthermore, the analysis, including the development of an analysis plan, was undertaken by an academic group, independent of the group responsible for the campaign. The analysis considered both the detailed trends over time and specific pre–post comparisons, with specific time-points chosen to account for a lesser/greater delay between the campaign and change in some metrics. We do acknowledge that most metrics were targeted at measuring the impact on lung cancer and so may not have fully captured any effect on other respiratory and cardiovascular diseases.

Data on awareness from the survey were arguably more open to bias than the routine data. Differences in the survey method between the 50–69 and the 70+ year age groups (online and face to face, respectively) increased the complexity of interpretation, accentuated further by data from 2016 face-to-face surveys having to be omitted from the analysis in order to make the survey methods consistent between 2016 and 2017. Furthermore, the surveys are perforce hypothetical, whereas all other metrics measured actual interaction with healthcare. On balance, we did not feel these compromised validity greatly, particularly as our conclusions did not rely on the survey results alone, with the downstream metrics supporting diagnostic improvements. Another important limitation is the design cannot differentiate the effects of the campaign from other changes occurring during the period of observation. Other initiatives to expedite diagnosis of cancer in symptomatic patients operated in England over the campaign, including initiatives encouraging GPs to investigate more, notably the NICE guidance published in June 2015 [23]. The continuing upward trend in lung cancer incidence itself could have contributed to changes and there are general upward trends in 2-week referral waits for cancer [24]. Overdiagnosis is a concern for lung cancer screening [25], raising the possibility that not all lung cancers identified may have been the cause of the symptoms. This ‘serendipitous’ percentage is estimated to be 27–48% [26].

Concerning other evaluations, Ironmonger et al. remain the main published evaluation of a national mass media cancer symptom awareness campaign, describing the first campaign for cough alerting to lung cancer in England, which ran in 2012 [12]. It was widely deemed to have been effective, although less so when the cough campaign was repeated in 2013 and 2014 [27]. Similar metrics were measured to here, but with a striking difference in an increase in GP attendances for cough in those aged >50 of 63%. It is interesting also that the 1-year 1.5% increase in cancer diagnoses in the ‘control’ period in Ironmonger et al. is similar to the 2-year 4% increase in this study and that both are very different from the 1-year 9.1% increase for the 2012 ‘campaign’ period. Other evaluations of local campaigns around the first national cough campaign have reported positive results [28, 29]. These combined public awareness campaigns with brief intervention GP training. Finally, an evaluation of a 4-week national campaign in Wales in 2016 at the same time as the first phase of the English campaign reported here showed improvements in awareness and attitudes with statistically significant increases in GP visits and GP-ordered chest X-rays, but without statistically significant changes in urgent referrals or lung cancer diagnoses [30].

This evidence offers a picture of varying effectiveness. All campaigns achieved their immediate objective of increasing awareness, but only some achieved directly attributable change in downstream healthcare metrics. It is tempting to dismiss this as merely the effect of the duration and intensity of the campaigns, but this view is challenged by the campaign we report here being longer than the 2012 campaign, which produced a positive effect on outcomes. Differences in the precise nature of the campaigns are likely to be important; for instance, the extension of the campaign beyond promoting referral for lung cancer alone to promoting referral for other serious diseases by the inclusion of shortness of breath as a symptom. The effect of national mass media cancer symptom awareness campaigns on health outcomes is a complex interaction with activity in other parts of the health service and the true complexity of this may not yet be fully understood. One particular aspect is the interaction between public awareness and primary care attendance, such as a partial failure of increased awareness to translate into increased attendances. A perceived lack of capacity may inhibit patients presenting to primary care, and although they may still engage with the health service via other routes, these are unlikely to be able to request a chest X-ray.

In conclusion, our pre–post study of a national mass media cancer awareness campaign targeting respiratory symptoms showed small positive results in the metrics most linked to survival—and in survival itself. A 2.11% improvement in early stage at diagnosis approximates to 1000 UK lung cancer sufferers becoming potentially curable each year and aligns well with the aspiration in the NHS Ten Year Plan to increase early stage at diagnosis. A benefit of this size suggests the BCOC campaign was a ‘success’—although this needs to be balanced against some uncertainty whether the changes can be wholly attributed to the campaign itself. The pattern over time challenges whether the improvements are attributable to the campaign. Greater changes relative to the background trend/pattern would have been expected in and around the campaign phases if this were the case, particularly on metrics like GP attendance and referrals, and this was not observed. Given the importance of education on cancer in its own right, raising awareness of cancer symptoms should remain an important approach. We need a better understanding of the interplay between public awareness and how that is translated into action (or not) by patients, GPs, specialists and the healthcare system. Redesign of campaigns [31], integration of them with other interventions [27], using different interventions to encourage appropriate response to possible lung cancer symptoms [32] and targeting activity to particular groups [33, 34] could all be more rationally deployed to maximise effectiveness and cost-effectiveness if there were a better theoretical model of action building on the updated NAEDI model [11].

## Supplementary information

Supplementary Table 1

Supplementary Table 2

Supplementary Table 3

Supplementary Figure 1

Supplementary Figure 2

Supplementary Figures 3 to 10

Reproducibility checklist
