# Supplementary material for: An evaluation of a national mass media campaign to raise public awareness of possible lung cancer symptoms in England in 2016 and 2017
Source: Br J Cancer. 2021 Oct 30;126(2):187–95. doi: 10.1038/s41416-021-01573-w (PMC8770501; doi:10.1038/s41416-021-01573-w)
Supplement: Supplementary file 1 — Supplementary Table 1 [file 41416_2021_1573_MOESM1_ESM.docx]

Supplementary Table 1: Changes in metrics for Phase 1 of the campaign in 2016

| **Metric** | **Comparison period**  **(pre Phase 1)** | **Analysis period**  **(during/post Phase 1)** | **Statistic** | **Estimate (95% CI)** | **p value** |
| --- | --- | --- | --- | --- | --- |
| GP attendances (people aged ≥50; visits with respiratory symptoms)* | 38,763.75 across  3,973 practices | 27,360.75 across  2,830 practices | Rate ratio | 0.99 (0.90 to 1.09) | 0.8 |
| TWW referrals | 24,066 | 25,925 | Rate ratio | 1.08 (1.01 to 1.15) | 0.02 |
| Cancer diagnoses resulting from a TWW referral [based on ‘date first seen’ in CWT database] | 4,401 | 4,547 | Rate ratio | 1.03 (0.99 to 1.08) | 0.1 |
| TWW referrals resulting in a cancer diagnosis (conversion rate: %) [based on ‘date first seen’ in CWT database] | 18.28  (4,401 out of 24,074) | 17.51  (4,545 out of 25,959) | Difference in  percentage | -0.77% (-1.45% to -0.10%) | 0.02 |
| Total new cancers recorded in CWT database [based on ‘treatment start date’ in CWT database] | 12,211 | 12,924 | Rate ratio | 1.06 (1.01 to 1.11) | 0.03 |
| Cancers diagnosed recorded in CWT database from TWW referral (detection rate: %) [based on ‘treatment start date’ in CWT database] | 37.62  (4,594 out of 12,211) | 36.54  (4,722 out of 12,924) | Difference in  percentage | -1.09% (-2.28% to 0.11%) | 0.08 |
| Emergency presentations [from inpatient HES] | 4,499 out of 12,711  (35.39%) | 4,446 out of 13,109  (33.92%) | Difference in  percentage | -1.48% (-2.64% to -0.32%) | 0.01 |
| Cancers diagnosed* [from cancer registration database] | 15,401.5 | 16,052.5 | Rate ratio | 1.04 (1.01 to 1.07) | 0.003 |
| Early stage at diagnosis* | 5,429.75 early from 14,099  staged (38.51%) | 5,995.25 early from 14,883  staged (40.28%) | Difference in  percentage | 1.77% (0.65% to 2.90%) | 0.002 |
| Diagnostics in secondary care: X-rays and CT scans | 920,970 images | 1,026,305 images | Rate ratio | 1.11 (1.03 to 1.20) | 0.006 |
| Echocardiograms | 577,736 echocardiograms | 619,885 echocardiograms | Rate ratio | 1.07 (1.02 to 1.13) | 0.005 |
| Outpatient attendances to cardiac or respiratory | 4,388,159 attendances | 4,511,786 attendances | Rate ratio | 1.03 (0.99 to 1.07) | 0.19 |
| Inpatient admissions to cardiac or respiratory | 417,614.8 admissions | 443,838.8 admissions | Rate ratio | 1.06 (1.02 to 1.11) | 0.004 |
| Major resections | 2,830 out of 18,698  (15.14%) | 3,077 out of 19,205  (16.02%) | Difference in  percentage | 0.89% (0.16% to 1.62%) | 0.02 |
| Survival (people aged ≥50) | Median survival 207 days | Median survival 217 days | Hazard ratio | 0.95 (0.93 to 0.98) | 0.002 |

* where numbers of cases are not whole numbers this is because weekly numbers of cases have been adjusted for bank holidays and summed over the period
