# Supplementary material for: An evaluation of a national mass media campaign to raise public awareness of possible lung cancer symptoms in England in 2016 and 2017
Source: Br J Cancer. 2021 Oct 30;126(2):187–95. doi: 10.1038/s41416-021-01573-w (PMC8770501; doi:10.1038/s41416-021-01573-w)
Supplement: Supplementary file 2 — Supplementary Table 2 [file 41416_2021_1573_MOESM2_ESM.docx]

.

Supplementary Table 2: Sensitivity analyses: changes in metrics across the whole campaign (Phases 1 and 2 combined), based on people aged ≥50.

| **Metric** | **Comparison period**  **(pre Phase 1)** | **Analysis period (during/post Phase 2)** | **Statistic** | **Estimate (95% CI)** | **p value** |
| --- | --- | --- | --- | --- | --- |
| TWW referrals | 21,479 | 23,727 | Rate ratio | 1.10 (1.02 to 1.20) | 0.01 |
| Cancer diagnoses resulting from a TWW referral [based on ‘date first seen’ in CWT database] | 4,388 | 4,422 | Rate ratio | 1.01 (0.95 to 1.07) | 0.8 |
| TWW referrals resulting in a cancer diagnosis (conversion rate: %) [based on ‘date first seen’ in CWT database] | 20.43 (4,388 out of 21,479) | 18.64 (4,422 out of 23,727) | Difference in percentage | -1.79% (-2.52% to -1.06%) | <0.001 |
| Total new cancers recorded in CWT database [based on ‘treatment start date’ in CWT database] | 12,459 | 13,362 | Rate ratio | 1.07 (1.02 to 1.13) | 0.01 |
| Cancers diagnosed recorded in CWT database from TWW referral (detection rate: %) [based on ‘treatment start date’ in CWT database] | 38.61 (4,811 out of 12,459) | 34.75 (4,643 out of 13,362) | Difference in percentage | -3.87% (-5.04% to -2.69%) | <0.001 |
| Emergency presentations [from inpatient HES] | 4,389 out of 12,330 (35.60%) | 4,348 out of 12,771 (34.05%) | Difference in percentage | -1.55% (-2.73% to -0.37%) | 0.01 |
| Cancers diagnosed* [from cancer registration database] | 15,963.75 | 16,564 | Rate ratio | 1.04 (1.02 to 1.06) | 0.001 |
| Early stage at diagnosis | 5,652 early staged out of 14,660 staged (38.55%) | 6,305 early staged out of 15,488 staged (40.71%) | Difference in percentage | 2.16% (1.05% to 3.26%) | <0.001 |
| Diagnostics in secondary care: X-rays and CT scans | 679,875 images | 784,735 images | Rate ratio | 1.15 (1.08 to 1.23) | <0.001 |
| Echocardiograms | DATA NOT AVAILABLE | | | | |
| Outpatient attendances | 2,890,654 | 3,091,789 | Rate ratio | 1.07 (1.05 to 1.09) | <0.001 |
| Inpatient admissions* | 323,061.3 | 355,975.5 | Rate ratio | 1.10 (1.07 to 1.13) | <0.001 |
| Major resections | 2,287 out of 15,509 (14.75%) | 2,657 out of 15,856 (16.76%) | Difference in percentage | 2.01% (1.20% to 2.82%) | <0.001 |

* where numbers of cases are not whole numbers this is because weekly numbers of cases have been adjusted for bank holidays and summed over the period
