# Supplementary material for: An evaluation of a national mass media campaign to raise public awareness of possible lung cancer symptoms in England in 2016 and 2017
Source: Br J Cancer. 2021 Oct 30;126(2):187–95. doi: 10.1038/s41416-021-01573-w (PMC8770501; doi:10.1038/s41416-021-01573-w)
Supplement: Supplementary file 3 — Supplementary Table 3 [file 41416_2021_1573_MOESM3_ESM.docx]

Supplementary Table 3: Sensitivity analyses: changes in metrics for Phases 1 of the campaign in 2016, based on people aged ≥50.

| **Metric** | **Comparison period**  **(pre Phase 1)** | **Analysis period (during/post Phase 1)** | **Statistic** | **Estimate (95% CI)** | **p value** |
| --- | --- | --- | --- | --- | --- |
| TWW referrals | 21,508 | 23,246 | Rate ratio | 1.08 (1.02 to 1.15) | 0.02 |
| Cancer diagnoses resulting from a TWW referral [based on ‘date first seen’ in CWT database] | 4,286 | 4,447 | Rate ratio | 1.04 (0.98 to 1.10) | 0.2 |
| TWW referrals resulting in a cancer diagnosis (conversion rate: %) [based on ‘date first seen’ in CWT database] | 19.93 (4,286 out of 21,508) | 19.13 (4,447 out of 23,246) | Difference in percentage | -0.80% (-1.53% to -0.06%) | 0.03 |
| Total new cancers recorded in CWT database [based on ‘treatment start date’ in CWT database] | 11,883 | 12,577 | Rate ratio | 1.06 (1.00 to 1.12) | 0.04 |
| Cancers diagnosed recorded in CWT database from TWW referral (detection rate: %) [based on ‘treatment start date’ in CWT database] | 37.64 (4,473 out of 11,883) | 36.72 (4,618 out of 12,577) | Difference in percentage | -0.92% (-2.14% to 0.29%) | 0.1 |
| Emergency presentations [from inpatient HES] | 4,389 out of 12,330 (35.60%) | 4,471 out of 12,587 (35.52%) | Difference in percentage | -0.08% (-1.26% to 1.11%) | 0.9 |
| Cancers diagnosed** [from cancer registration database] | 15,018.25 | 15,637.5 | Rate ratio | 1.04 (1.01 to 1.07) | 0.004 |
| Early stage at diagnosis | 5,313.5 early stage out of 13,747 staged (38.65%) | 5,875 early stage out of 14,492 staged (40.54%) | Difference in percentage | 1.89% (0.75% to 3.03%) | 0.001 |
| Diagnostics in secondary care: X-rays and CT scans | 671,785 images | 752,645 images | Rate ratio | 1.12 (1.05 to 1.19) | <0.001 |
|  |  | | | | |
| Outpatient attendances | 3,159,142 attendances | 3,264,661 attendances | Rate ratio | 1.03 (0.99 to 1.08) | 0.12 |
| Inpatient admissions | 342,394.3 admissions | 362,207 admissions | Rate ratio | 1.06 (1.02 to 1.09) | 0.001 |
| Major resections | 2,701 out of 18,218 (14.83%) | 3,101 out of 18,501 (16.76%) | Difference in percentage | 1.94% (1.19% to 2.68%) | <0.001 |

* where numbers of cases are not whole numbers this is because weekly numbers of cases have been adjusted for bank holidays and summed over the period
