# Supplementary material for: An evaluation of a national mass media campaign to raise public awareness of possible lung cancer symptoms in England in 2016 and 2017
Source: Br J Cancer. 2021 Oct 30;126(2):187–95. doi: 10.1038/s41416-021-01573-w (PMC8770501; doi:10.1038/s41416-021-01573-w)
Supplement: Supplementary file 4 — Supplementary Figure 1 [file 41416_2021_1573_MOESM4_ESM.docx]

Figure S1: GP attendances
