# Supplementary material for: An evaluation of a national mass media campaign to raise public awareness of possible lung cancer symptoms in England in 2016 and 2017
Source: Br J Cancer. 2021 Oct 30;126(2):187–95. doi: 10.1038/s41416-021-01573-w (PMC8770501; doi:10.1038/s41416-021-01573-w)
Supplement: Supplementary file 6 — Supplementary Figures 3 to 10 [file 41416_2021_1573_MOESM6_ESM.docx]

Figure S3: Cancer diagnoses resulting from urgent GP referral.

Figure S4: Cancers diagnosed recorded in CWT database.

Figure S5: Cancers diagnosed.

Figure S6: Diagnostics in secondary care: X-rays and CT scans.

Figure S7: Echocardiograms.

Figure S8: Outpatient attendances.

Figure S9: Inpatient admissions.

Figure S10: Major resections.
